# Platelet‐rich plasma and plasma rich in growth factors in extra‐oral wound care

**DOI:** 10.1111/prd.12572

**Published:** 2024-07-26

**Authors:** Jeniffer Perussolo, Elena Calciolari, Xanthippi Dereka, Nikolaos Donos

**Affiliations:** ^1^ Centre for Oral Clinical Research, Institute of Dentistry, Faculty of Medicine and Dentistry Queen Mary University of London London UK; ^2^ Department of Medicine and Surgery, Dental School University of Parma Parma Italy; ^3^ Department of Periodontology, School of Dentistry, Dental School National and Kapodistrian University of Athens Athens Greece

**Keywords:** extra‐oral, healing, plasma rich in growth factors, platelet‐rich plasma, wound

## Abstract

This narrative review evaluates the existing literature on the clinical efficacy and safety of platelet‐rich plasma (PRP) and plasma rich in growth factors (PRGFs) in extra‐oral wound care, considering their potential benefits and drawbacks. The review specifically focuses on the impact of these treatments on patients' quality of life, pain management, treatment costs, recurrence rates, and potential complications. Given the extensive literature and diverse range of extra‐oral wound types in which these autologous platelet concentrates have been applied, this narrative review focuses on the most frequently described wound types, including diabetic foot ulcers, venous leg ulcers, pressure ulcers, surgical wounds, and burns. The use of PRP has been reported in various medical specialties, with a low risk of adverse events. While there is a growing interest in the use of PRGF with promising results, the available literature on this topic is still limited. Only a few studies evaluated patients' perception of the treatment and the relationship between treatment costs and clinical outcomes. Data on recurrence rates and complications also vary across studies. In conclusion, PRP and PRGF show promise as alternatives or as adjunctive therapies to conventional treatments for various extra‐oral wounds and ulcers, leading to reduced wound size and accelerated healing time but should be considered on a case‐by‐case basis, taking into account the type and severity of the wound.

## 1 INTRODUCTION

A wound is a disruption in the normal structure and function of the skin, mucous membranes, or other body tissues, caused by a variety of factors, including trauma, burns, surgery, or underlying medical conditions.^1^, ^2^ The continuous increase in the global demand for wound care finds its roots in the escalating prevalence of chronic diseases, such as diabetes mellitus, and conditions that affect wound healing, as well as in the growing number of surgical procedures being performed.^3^, ^4^, ^5^

The wound healing is a complex and dynamic process that involves various biological and molecular events. These events include hemostasis, inflammation, proliferation, and tissue remodeling/maturation, which are regulated by a diverse range of cells as well as soluble biomarkers, such as growth factors and cytokines.^6^ Although both dermal wound healing and oral mucosal wound healing proceed through the same phases, dermal wounds tend to heal at a slower pace and often lead to scar formation.^7^ These differences may stem from inherent disparities in the molecular profile and cellular responses at the respective sites.^7^, ^8^ It has been shown that the levels of both inflammatory and pro‐fibrotic cytokines are higher in dermal tissues than in the oral mucosa, explaining the slower healing and scar formation.^7^ Nevertheless, the primary objective in both extra‐ and intra‐oral wound care is to achieve wound closure as promptly as possible, reducing the risk of complications and enhancing patients' quality of life. The medical literature has outlined four crucial factors that must be systematically addressed to promote wound healing: (1) removal of nonviable or necrotic tissue, foreign bodies, exudate, and/or biofilm; (2) identification of the wound's etiology, infection, and inflammation management; (3) application of adequate dressing to regulate exudate levels and maintain moisture balance; and finally (4) assessment of the wound edge, which reflects the progress of wound healing and confirms the efficacy of the applied therapy.^9^, ^10^

Despite the availability of a multitude of local wound care modalities, including but not limited to wound debridement, dressings, surgical management involving skin grafts and revascularization, and pharmacotherapeutics and medical devices (i.e., negative pressure wound therapy and lasers), healthcare providers still encounter significant challenges in achieving wound healing.^6^, ^11^ In the United Kingdom, between 2017 and 2018, approximately 3.8 million adult patients were affected by wounds, accounting for 7% of the adult population. Notably, during this period, the National Health Service (NHS) allocated a substantial sum of about £8.3 billion to manage these patients.^3^ According to the report, approximately 30% of all wounds (equivalent to 1.1 million cases) remained unhealed, leading to a significant increase in resource utilization and costs. In fact, the annual cost of managing unhealed wounds was estimated to be £5.6 billion.^3^ Meanwhile, in the United States, total spending estimates of the federal health insurance program (Medicare), in 2014, for all wound types ranged from $28.1 to $96.8 billion.^5^ As a result, the clinical and economic implications have led professionals to seek new and alternative strategies to improve the management of different types of wounds. These strategies aim to accelerate healing, reduce hospital stay and associated costs, improve esthetic outcomes, minimize complications (such as infection, recurrence, graft necrosis, and limb amputation), improve patients' quality of life, repair the structure and function of injured tissue, and lower mortality rates.

In recent years, there has been a growing interest in treatment options that not only promote wound closure but also enhance the body's natural healing processes. These adjunctive therapies, which can be used alongside conventional medical treatments, target the molecular mechanisms responsible for controlling cellular signaling pathways involved in tissue regeneration, while also reducing the inflammatory response.^12^ Platelet‐rich plasma (PRP) is an autologous or allogeneic derivative of whole blood that, after centrifugation, contains a high concentration of platelets suspended in a small amount of plasma. As a matter of fact, platelets play a fundamental role in mediating the healing of tissues due to their ability to release cytokines and growth factors, including platelet‐derived growth factor (PDGF), transforming growth factor‐beta (TGF‐β), vascular endothelial growth factor (VEGF), insulin‐like growth factor‐1 (IGF‐1), fibroblast growth factor (FGF), and epidermal growth factor (EGF), which promote cell migration, proliferation, and differentiation, as well as angiogenesis and collagen fiber synthesis.^13^, ^14^, ^15^, ^16^ Hence, over the years, there has been a rising body of research exploring the potential of PRP as an adjunctive therapy for extra‐oral wound care and its influence in the fields of tissue engineering and regenerative medicine with promising results. More recently, a derivative of PRP, the plasma rich in growth factor (PRGF), has also received interest, particularly in oral surgery and ophthalmology. PRGF is elaborated by a one‐step centrifugation process and is characterized by a more sustained release of growth factors as calcium chloride instead of thrombin is used. PRGF has a moderated platelet concentration and does not contain leukocytes, with the aim of avoiding the proinflammatory effects of the proteases and acid hydrolases contained in white blood cells.^17^, ^18^ However, fewer studies have been conducted on its use.

Therefore, this review aims to comprehensively evaluate the existing literature regarding the clinical efficacy and safety of PRP and PRGFs in extra‐oral wound care, as well as their potential benefits and drawbacks. The review also provides information about distinct types of extra‐oral wounds and their causes, investigating the impact of treatment on patients' quality of life (i.e., pain), cost implications, and potential complications.

## 2 MATERIALS AND METHODS

Despite this manuscript being a narrative review, the authors have developed a comprehensive search strategy (Appendix S1) that combined MeSH terms and free text in an attempt to identify all clinical studies that evaluated the efficacy of PRP or PRGF for the treatment of extra‐oral wounds. We searched two main databases, MEDLINE via Ovid and Embase for papers published from 1946 to January 13, 2023.

Due to the large number of papers on this topic and the wide range of extra‐oral wound types in which PRP has been applied, we limited our review to the most frequently described wound types, for which the largest evidence is available, including diabetic foot ulcers (DFUs), venous leg ulcer (VLU), pressure ulcers, surgical wounds, burns, and other wound types. To further narrow our review, we focused primarily on randomized controlled trials (RCTs), controlled clinical trials (CCTs), and prospective and retrospective studies that presented data on clinical efficacy and safety, patient‐related outcomes, complications, treatment costs, and morbidity. However, we also included and discussed case series, especially for PRGF use, whenever fewer clinical evidence was available. Pre‐clinical animal studies and case reports with fewer than five patients were excluded from this review.

## 3 PRP/PRGF APPLICATIONS IN EXTRA‐ORAL WOUND CARE IN HUMANS

The extra‐oral wounds can often be categorized based on their duration and underlying causes. Acute wounds are those that typically occur suddenly and have a rapid onset, such as burns, surgical wounds, and traumatic injuries.^3^, ^4^ Although the human body has a remarkable ability to heal itself, a significant number of wounds do not follow this pattern and tend to become chronic. Chronic wounds, also known as nonhealing wounds, are those that fail to progress within a specific timeframe or progress slowly through the stages of healing despite appropriate care,^19^, ^20^, ^21^, ^22^ leading to prolonged suffering,^2^ risk of infection, and increased medical expenses.^9^

**FIGURE 1:**
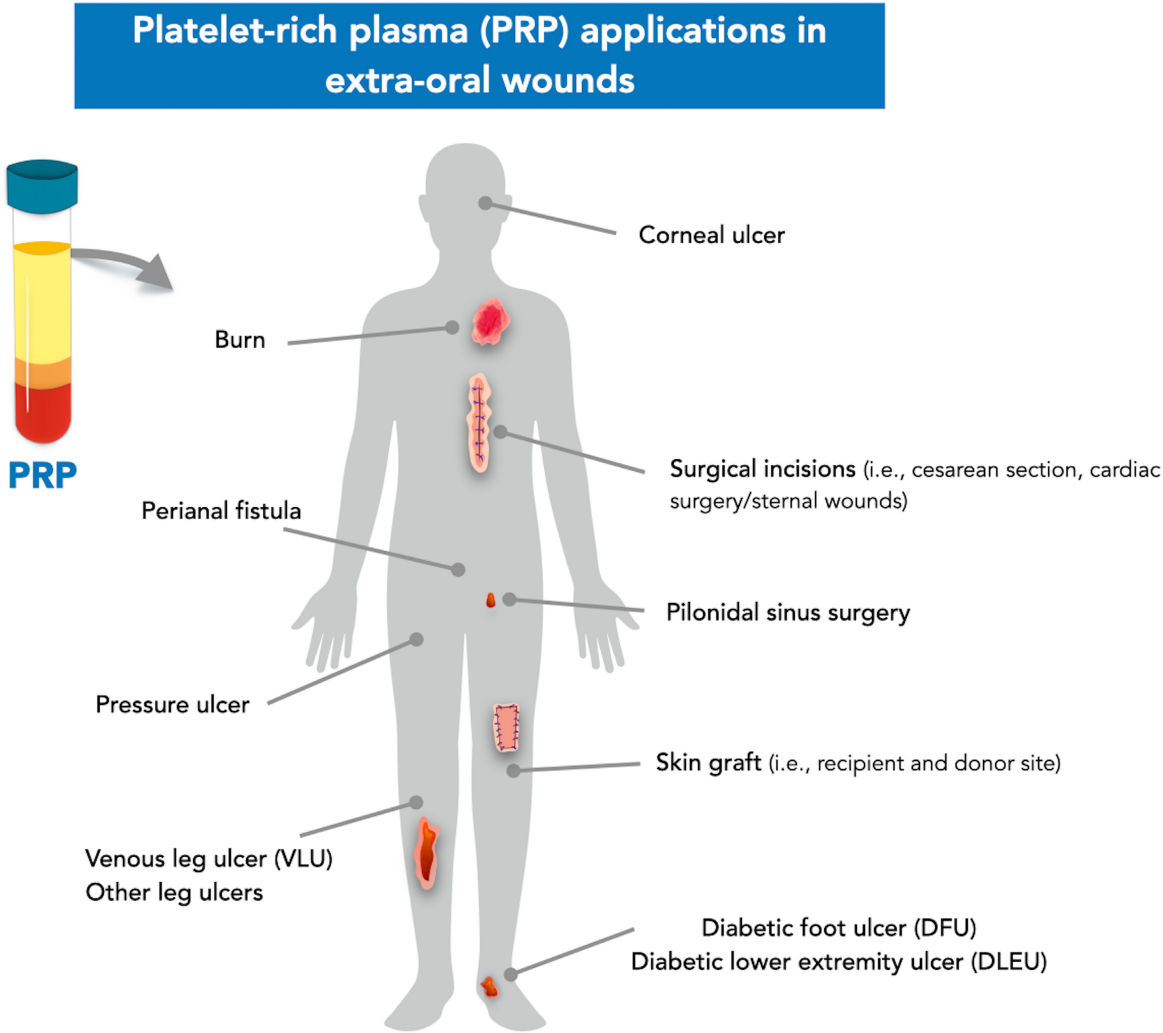
Platelet‐rich plasma (PRP) applications in extra‐oral wounds.

The use of PRP and its derivatives has been reported across various medical specialties, including orthopedics, plastic surgery, dermatology, ophthalmology, and cardiovascular surgery as an effective and safe option to accelerate the healing of extra‐oral wounds (Figure 1). Table 1 presents the main characteristics of the RCTs and CCTs included in this narrative review.

**TABLE 1:** Main characteristics of the RCTs and CCTs included in this narrative review.

Author (year)	Study type; follow‐up	Wound type	Treatment allocation (*n*. patients per group)	Wound size/healing time
**Chronic ulcers**				
Driver et al. (2006)	RCT; 3 months	DFU on the plantar, medial, or lateral aspect of the foot	**Control**: Wound debridement and application of a saline gel covered with a contact layer dressing (primary) and a foam dressing (secondary) (*n* = 21) **Test**: Wound debridement and application of PRP gel covered with a contact layer dressing (primary) and a foam dressing (secondary) (*n* = 19)	*Wound healing time (mean ± SD)* **Control**: 47.4 ± 22.0 days **Test**: 42.0 ± 18.3 days
Anitua et al. (2008)	Pilot RCT; patients followed until either complete healing (full epithelization) or 8 weeks of treatment	Nonhealing ulcer of less than 12 cm in diameter, present for >4 weeks 14 ulcers assessed (64% VLU, 29% pressure ulcer, and 7% other)	**Control**: Standard wound care: debridement (if infection suspected), wound cleaning with saline, and moist saline gauze dressing (secondary) (*n* = 5) **Test**: Standard wound care and PRGF (*n* = 4)	*Surface healed at 8 weeks (mean% ± SD)* **Control**: 21.5% ± 33.6 **Test**: 72.9% ± 22.3
Ramos‐Torrecillas et al. (2015)	RCT; 36 days	Pressure ulcer for more than 8 weeks	**Control**: Standard care: ulcer debridement, cleaning with saline and sterile gauze, application of liquid hydrogel, and placement of a polyurethane dressing (*n* = 25 PUs) **Test 1**: Standard care plus topical application of one dose of PRGF (*n* = 34 PUs) **Test 2**: Standard care plus topical application of two doses of PRGF (*n* = 25 PUs) **Test 3:** Standard care plus topical application of two doses of PRGF and HA (*n* = 40 PUs)	*Reduction of area of ulcer (mean% ± SD)* **Control**: 10.3% ± 13.3 **Test** **1**: 48.3% ± 25.8 **Test** **2**: 54.8% ± 44.7 **Test** **3**: 80.4% ± 27.0
Cardeñosa et al. (2016)	RCT; 24 weeks	Venous ulcers of more than six weeks' clinical course	Prior to randomization all patients underwent cleaning and mechanical debridement of ulcer **Control**: Gauze soaked in saline, dry gauze (secondary dressing), and single‐layer pressure bandage (*n* = 47) **Test**: PRGF covered with a selective micro‐adherence dressing (primary dressing), gauze (secondary dressing), and single‐layer pressure bandage (*n* = 55)	*Area of the ulcer (mean ± SD) and percentage healed area (mean %)* **Control**: *Area*: 16.7 ± 23.9 cm^2^ before intervention; 12.1 ± 19.2 cm^2^ after intervention *Healed area*: 11.2% ± 24.4 **Test**: *Area*: 13.7 ± 30 cm^2^ before intervention; 9.9 ± 29.9 cm^2^ after intervention *Healed area*: 67.7% ± 41.5
Burgos‐Alonso et al. (2018)	Pilot RCT; 9 weeks	VLUs present for more than 8 weeks without response to conventional treatment	**Control**: Standard care, waterproof polyurethane dressing, and compression therapy (*n* = 3) **Test**: Cleaning, mechanical debridement, PRP gel application, secondary dressing, and compression therapy (*n* = 5)	*Reduction of area of ulcers at 9 weeks (mean %)* **Control**: 40.8% **Test**: 82.8%
Elbarbary et al. (2020)	RCT; up to 12 months	VLUs for at least the last 6 weeks	**Control**: Compression therapy alone (*n* = 30) **Test 1**: PRP dressing application, a nonabsorbent dressing, and compression therapy with single layer of elastic bandage (*n* = 30) **Test 2**: Local PRP injection inside the ulcer, a nonabsorbent dressing, and compression therapy with single layer of elastic bandage (*n* = 30)	Wound healing time (median) and reduction of area (mean %) **Control**: *Healing time*: 7 months *Area reduction*: 52% at 3 months; 69% at 6 months; 93% at 12 months **Test 1**: *Healing time*: 5 months *Area reduction*: 65% at 3 months; 92% at 6 months; 92.7% at 12 months **Test 2**: *Healing time*: 3 months *Area reduction*: 84% at 3 months; 90% at 6 months; 93% at 12 months
He et al. (2020)	CCT; participants were followed until wound closure was complete or until surgical operation, even amputation	DLEU	**Control**: Standard wound care: Surgical debridement, drainage with topical dressing, or negative pressure wound therapy (*n* = 30) **Test 1**: Standard wound care plus autologous platelet‐rich plasma (au‐PRP) (*n* = 25) **Test 2**: Standard wound care plus allogeneic platelet‐rich plasma (al‐PRP). An ABO‐ and Rh‐matched platelet concentrate was ordered from the blood bank (*n* = 20)	*Wound healing time (mean ± SD)* **Control**: 88.0 ± 43.4 days **Test 1**: 55.6 ± 33.8 days **Test 2**: 56.9 ± 29.2 days
Uçar and Çelik (2020)	RCT; 2 months	Coccyx pressure ulcer	**Control**: Ulcer cleaned with serum physiologic, covered with sterile gauze, and fixed with cotton tapes (*n* = 30) **Test**: Ulcer cleaned with saline, covered with sterile gauze, impregnated with PRP gel, and fixed with cotton tapes (*n* = 30)	*PUSH score (mean ± SD)* **Control**: 9.5 ± 2.2 at baseline; 9.4 ± 2.4 at 2 month (20th observation) **Test**: 8.4 ± 2.3 at baseline; 5.0 ± 3.9 at 20th observation
Alamdari et al. (2021)	RCT; 6 months	DFU on the medial, lateral, dorsal, or plantar aspect of the foot, lasting more than 4 weeks	**Control**: Wound debridement, irrigation with saline, and dressing with silver sulfadiazine ointment (*n* = 47) **Test**: Wound debridement and PRP (*n* = 43)	*Wound healing time (mean ± SD)* **Control**: 80 ± 11.4 days **Test**: 55 ± 10.4 days
Helmy et al. (2021)	RCT; up to 12 months	VLU located in the lower third of the leg	**Control**: Cleaning using normal saline, nonadherent dressing, and compression (*n* = 40) **Test**: Intradermal and subdermal injection of PRP in all edges and in the granular floor of the ulcer (*n* = 40)	*Wound healing time (mean ± SD) and reduction of area (mean %)* **Control**: *Healing time*: 4.7 ± 4.1 months *Area reduction*: 80.8% at 3 months; 89.4% at 6 months; 82.6% at 12 months **Test**: *Healing time*: 2.13 ± 0.5 months *Area reduction*: 94.8% at 3 months; 89.4% at 6 months; 82.6% at 12 months
Liu et al. (2021)	RCT; 21 days after treatment	Refractory pressure injuries	**Control**: Negative pressure wound therapy (*n* = 51) **Test**: Negative pressure wound therapy plus PRP gel (*n* = 51)	*Clinical efficacy (%)* **Control**: 39/51 (76.47%) **Test**: 47/51 (92.16%)
Singh et al. (2021)	RCT; 6 weeks	Sacral pressure ulcer	**Control**: Cleaning with saline, debridement, application of hydrogel all over the wound, and covered with sterile cotton gauze (*n* = 26) **Test**: Cleaning with saline, debridement, PRP injected into the ulcer margin and base, and covered with the sterile cotton gauze (*n* = 26)	*Wound surface area (mean)* **Control**: 36.4 cm^2^ at baseline; 25.9 cm^2^ at 3 weeks; 15.7 cm^2^ at 6 weeks **Test**: 37.0 cm^2^ at baseline; 25.9 cm^2^ at 3 weeks; 14.6 cm^2^ at 6 weeks
Shehab et al. (2023)	RCT; patients followed weekly (6 times of application + end‐visit)	Chronic post‐phlebitic lower limb venous ulcers	**Control**: Placebo and compression therapy (*n* = 20) **Test:** PRP, nonabsorbent dressing, and compression therapy (*n* = 20)	*Reduction of area and volume of ulcers* *(mean %)* **Control**: *Area reduction*: 40% *Volume reduction*: 48% **Test**: *Area reduction*: 74% *Volume reduction*: 81%
**Surgical wounds**				
Tehranian et al. (2016)	RCT; 8 weeks	Cesarean section wound	**Control**: No topical treatment; the subcutaneous tissue was cleaned with normal saline (*n* = 71) **Test**: PRP was directly applied to the subcutaneous tissue of the wound site (*n* = 67)	*REEDA score (mean ± SD)* **Control**: 2.5 ± 0.6 at day 1, 1.9 ± 0.6 at day 5, 0.9 ± 0.5 at week 8 **Test**: 2.4 ± 0.7 at day 1, 1.3 ± 0.6 at day 5, 0.7 ± 0.5 at week 8
Guerid et al. (2017)	RCT; patients followed up to complete healing	Donor site in patients requiring harvesting of STSG	**Control**: Three layers of paraffin gauze and bandages (*n* = 15) **Test 1**: PRP and three layers of paraffin gauze and bandages (*n* = 15) **Test 2**: Suspension of keratinocytes, PRP, and three layers of paraffin gauze and bandages (*n* = 15)	*Wound healing time (mean ± SD)* **Control**: 13.9 ± 0.5 days **Test 1:** 7.2 ± 0.2 days **Test 2**: 5.7 ± 0.2 days
Hersant et al. (2017)	Pilot RCT; patients followed until complete healing was achieved	Necrotizing fasciitis or Fournier's gangrene treated with STSG	**Control**: Debridement and excision of necrotic tissue and STSG (*n* = 13) **Test**: Debridement of necrotic tissue, STSG + A‐PRP/thrombin gel (*n* = 14)	*Complete healing time (mean ± SD)* **Control**: 73.7 ± 50.8 days **Test**: 37.9 ± 14.3 days
Slaninka et al. (2020)	RCT (one side test and one side control); patients followed up to complete healing	Donor site in patients requiring dermo‐epidermal skin graft	**Control**: Vaseline‐impregnated open‐weave gauze (first layer) and gauze squares (second layer) (*n* = 24) **Test**: Vaseline‐impregnated open‐weave gauze (first layer) and gauze squares (second layer) + PRP (*n* = 24)	*Wound healing time (mean)* **Control**: 18.4 days **Test 1**: 14.9 days
Elkhouly et al. (2021)	RCT; 6 months	Cesarean section wound	**Control**: No topical treatment; the subcutaneous tissue was cleaned with normal saline (*n* = 96) **Test**: PRP was directly applied to the subcutaneous tissue of the wound site (*n* = 98)	*REEDA score (mean ± SD)* **Control**: 5.1 ± 1.2 at day 1, 3.7 ± 1.4 at day 7, 2.5 ± 1.1 at 6 months **Test**: 4.2 ± 1.1 at day 1, 2.6 ± 1.0 at day 7, 1.5 ± 0.9 at 6 months
Jain et al. (2021)	CCT^a^ (mesial half of wound test – distal half wound control); 21 days	Donor site in patients requiring harvesting of STSG	**Control**: Distal half of wounds covered with petroleum gauze dressing (*n* = 20) **Test**: PRP sprayed on proximal half of donor site and covered with petroleum gauze dressing (*n* = 20)	*Complete healing at 14 and 21 days* **Control**: *14 days*: 0/20 *21 days*: 19/20 **Test**: *14 days*: 11/20 *21 days*: 19/20
Menchisheva et al. (2021)	RCT; 90 days	Plastic and reconstructive surgery in the maxillofacial area	**Control**: No injection (*n* = 50) **Test**: PRP intradermally injected, 0.5 cm from the edge of the wound; remaining plasma applied on the postoperative wound (*n* = 50)	*Scar width (median)* **Control**: 2 mm at 3.5 days, 2 mm at day 7, 2 mm at day 10, 3 mm at day 30 **Test**: 1 mm at 3.5 days, 1 mm at day 7, 1 mm at day 10, 1.5 mm at day 30
Wang et al. (2021)	CCT; patients followed up to fracture healing time	Open fractures with soft tissue defects treated with skin flap transplantation	**Control**: Debridement, removal of necrotic tissue, fracture reduction, and skin transplant (*n* = 35) **Test**: Debridement, removal of necrotic tissue, fracture reduction PRP, and skin transplant (*n* = 37)	*Wound healing time (mean ± SD)* **Control**: 32.2 ± 3.3 days **Test**: 22.4 ± 2.1 days Lower wound volume at 3 and 6 weeks reported in the test group (no raw data provided)
Ali et al. (2022)	CCT; 21 days	Donor site in patients requiring harvesting of STSG	**Control**: Distal half of wounds covered with petroleum gauze dressing (*n* = 15) **Test**: PRP sprayed on proximal half of donor site and covered with petroleum gauze dressing (*n* = 15) PRP followed by paraffin gauze	*Remaining raw area (%)* **Control**: 68.5% at day 7, 30.4% at day 14, 0.7% at day 21 **Test**: 44.5% at day 7, 16% at day 14, 0.2% at day 21
Gupta et al. (2022)	RCT; 21 days	Donor site in patients requiring harvesting of STSG	**Control**: Paraffin gauze dressing at donor site (*n* = 50) **Test**: PRP at the donor site followed by the standard paraffin gauze dressing (*n* = 50)	*Complete healing at 14 days* **Control**: 48/50 **Test**: 35/50
**Burns**				
Kazakos et al. (2009)	RCT; 6 months	Wide friction burns in the femur	**Control**: Topical washing and cleaning (*n* = 32) **Test**: Local application of PRP gel (*n* = 27)	*Wound healing time (mean ± SD)* **Control**: 40.6 ± 5.3 days **Test**: 21.3 ± 1.4 days
Maghsoudi et al. (2013)	RCT; mean follow‐up 16 weeks (range: 12–18)	Grade II and III burn wounds	**Control**: Silver sulfadiazine (*n* = 25) **Test**: Platelet concentrate (*n* = 25)	*Wound healing time (mean ± SD)* **Control**: 12.2 ± 5.4 days **Test**: 9.5 ± 4.6 days
Marck et al. (2016)	RCT (in each patient, two comparable wound areas); 5–7 days	Full‐thickness or deep dermal burn wound with a surface area of at least 2% total body surface area (TBSA)	**Control**: Split‐thickness skin graft (*n* = 49) **Test**: Split‐thickness skin graft plus PRP (*n* = 49)	*Graft take and epithelialization* **Control**: *Graft take*: 78.9% ± 25.1 *Epithelialization*: 67% ±29.1 **Test**: *Graft take*: 80.9% ± 25.5 *Epithelialization*: 69.5% ± 29.3
Liu et al. (2018)	RCT; 1 month	Deep grade II burn	**Control**: Debridement, cleaning, Silvadene cream by external application covered with 10 layers of sterile gauze (*n* = 34) **Test**: Debridement, cleaning, application of autologous platelet‐rich gel (APG), covered with 10 layers of sterile gauze (*n* = 34)	*Wound healing time (mean ± SD) and ratio of healed area (mean% ± SD)* **Control**: *Healing time*: 20.7 ± 6.6 days *Ratio of healed area*: 77.1% ± 10.4 days **Test**: *Healing time*: 16.8 ± 5.7 days *Ratio of healed area*: 84.8% ± 12.5 days

Abbreviations: CCT, controlled clinical trial; DFUs, diabetic foot ulcers; DLEUs, diabetic lower extremity ulcers; PRGF, plasma rich growth factor; PRP, platelet‐rich plasma; PUSH score, area of pressure wound, amount of exudate, and tissue type; RCT, randomized clinical trial; REEDA, Redness, Edema, Ecchymosis, Discharge, Approximation; STSG, split‐thickness skin graft; VLU, venous leg ulcer.

^a^ Study reclassified as CCT despite declared as RCT; clinical efficacy – number of sites in which wound surface was completely covered by epithelium and fresh granulation tissue or in which purulent secretions from the wound reduced, the wound size was reduced by more than 25%, and if fresh granulation tissue appeared after 21 days of treatment.

### 3.1 Chronic wounds

Chronic wounds are often associated with underlying medical conditions such as diabetes, venous or arterial insufficiency, or local disorders (i.e., persistent localized pressure)^3^, ^4^ and frequently affect the body lower extremities. These wounds can persist for weeks, months, or even years and require specialized care and management to facilitate healing.

One of the main causes of chronic nonhealing ulcer is diabetes mellitus. DFUs also known as diabetic lower extremity ulcers (DLEUs) are usually located on the medial, lateral, dorsal, or plantar aspect of the foot.^21^ These ulcers may develop as a consequence of the sustained effect of peripheral neuropathy, poor blood supply, and the increased risk of infections, together with injuries to pressure points.^23^ Furthermore, the hyperglycemia in diabetic patients may affect the neutrophil function, which will release factors capable to degrade the extracellular matrix and biomarkers involved in wound healing.^23^

Another common type of chronic wound is the leg ulcer, whose main presentation is the VLU^24^ caused by venous insufficiency.^11^ Venous leg ulcers (VLUs) resist healing in 30% and recur in 70% within 5 years of compression therapy alone.^11^ Pressure ulcers (PUs) or bedsores, defined as damage to the skin and/or underlying tissue induced either by prolonged or repetitive pressure alone or by a pressure–shear combination, can also be considered as hard‐to‐heal wounds.^25^ These injuries often occur in patients on long‐term bed rest and those with difficulty moving their lower extremities, due to hypoxia/ischemia resulting from long‐term compression, which leads to tissue necrosis.^12^, ^25^

These types of wounds tend to affect elderly patients that may also present other systemic comorbidities (malnutrition, infection, anemia, skin problems, and immune deficiency) and take medication such as antiplatelet drugs, which can affect healing. Description of chronic wounds and conventional treatment frequently offered is presented in Table 2.

**TABLE 2:** Description of chronic wounds and conventional treatment frequently offered.

Type of chronic wound	Description	Standard of care or conventional therapy^a^
Diabetic foot ulcer (DFU)	Ulcers usually located on the medial, lateral, dorsal, or plantar aspect of the foot,^21^ which may develop in diabetic patients as a consequence of the sustained effect of peripheral neuropathy, poor blood supply, and the increased risk of infections, together with injuries to pressure points^23^	*Systemic management* ^26^ Control of blood sugar, blood pressure, and blood lipids Nerve‐trophic and circulation‐improving therapies, if necessary Nutritional support Pain and infection control. Systemic antibiotic, where clinical signs of infection are present^26^ *Topical wound care* Removal of nonviable tissue/debridement^36^ Cleaning with saline/serum physiologic or other Dressing (e.g., silver sulfadiazine ointment dressing^21^ and saline gel^20^) Drainage with topical dressing or negative pressure wound therapy^26^ Offloading of the ulcer (relieving pressure)^36^
Venous leg ulcer (VLU)	A VLU is a break in the skin below the knee caused by sustained venous hypertension, which results from chronic venous insufficiency^11^, ^24^ due to venous valve incompetence or an impaired calf muscle pump^95^	*Systemic management* Pain and infection control. Systemic antibiotic, where clinical signs of infection are present^11^, ^31^ Lifestyle advice to promote ulcer healing and reduce the risk of recurrence, for example, encouraging elevating legs^29^ when immobile and avoiding leg trauma Use of medication to increase microcirculatory blood flow and improve ulcer healing^96^ Managing associated conditions, such as edema and venous eczema *Topical wound care* Removal of nonviable tissue/debridement^31^ Cleaning with saline/serum physiologic, chlorhexidine soap, or other to control microbial burden^31^ Dressing (e.g., Vaseline gauze^11^ and polyurethane^29^, ^30^) Compression therapy to facilitate return circulation^11^, ^29^, ^30^, ^31^, ^97^
Pressure ulcers (PUs) or bedsores	Damage to the skin and/or underlying tissue induced either by prolonged or repetitive pressure alone or by a pressure–shear combination.^25^ Common in patients on long‐term bed rest and those with difficulty moving their lower extremities, due to hypoxia/ischemia resulting from long‐term compression, which leads to tissue necrosis^12^, ^25^	*Systemic management* Evaluate the individual's comorbidities and promote disease control Nutritional support^25^ Pain and infection control. Systemic antibiotic, where clinical evidence of systemic infection *Topical wound care* Removal of nonviable tissue/debridement^12^, ^33^, ^35^, ^41^ Cleaning with saline/serum physiologic or other; use of topical antiseptics to control microbial burden^25^ Dressing (e.g., hydrocolloid, foam, polyurethane,^35^ and hydrogel^25^, ^32^) Biophysical agents such as negative pressure wound therapy^12^ Reposition of the individual and offloading strategies (such as mattresses and wheelchair cushions) of all bony prominences and maximum redistribution of pressure^25^

^a^ Type of conventional treatment offered may vary among health centers and studies. The correct dressing for wound management will depend not only on the type of wound but also on the stage of the healing process, which is beyond the scope of this review.^98^

#### 3.1.1 Wound size and healing

##### Diabetic foot ulcer

A recent meta‐analysis, including 8 controlled studies with moderate risk of bias, showed that PRP treatment in patients with DFU increased the likelihood of wound healing and decreased the volume of the ulcer compared to standard treatment, while reducing the time to complete healing.^6^ One of the studies included in this meta‐analysis showed that PRP significantly increased the healing rate and reduced the mean number of days to heal a DFU when compared to irrigation with saline and silver sulfadiazine ointment dressing (55 ± 10.4 vs. 80 ± 11.4 days) regardless of age, gender, smoking, and blood pressure status of patients.^21^ Remarkably, a prospective, randomized, controlled trial has shown that in the most common DFU size (≤7.0 cm^2^ in area and ≤2.0 cm^3^ in volume), although both PRP and saline gel presented a similar rate for wound area closure per day and healing period of approximately 6 weeks, 81.3% of PRP gel‐treated wounds and 42.1% of control gel‐treated wounds healed during that time.^20^

Despite its clinical benefits, autologous PRP (au‐PRP) preparations are variable and may present some clinical limitations, in particular, in patients with poor physical (i.e., platelet deficiency or disease, bleeding disorders, severe infection, and long‐term use of antiplatelet drugs) and mental conditions or in the treatment of wounds which require harvesting of larger quantities of blood.^26^ Therefore, a study by He et al. investigated and compared the effectiveness and safety of allogeneic platelet‐rich plasma (al‐PRP) from a blood bank to au‐PRP and conventional wound care in the treatment of 75 inpatients with DLEUs.^26^ The healing time of ulcers in the al‐PRP group (56.9 ± 29.2 days) and the au‐PRP group (55.6 ± 33.8 days) was equivalent and significantly shorter than that of ulcers in the control group (88.0 ± 43.4 days).^26^ However, al‐PRP preparation requires a strict process of sterilization and the exclusion of infectious disease before use.^26^

Although there are numerous studies on the use of PRP for DFU treatment, there is currently limited research on the use of PRGF in this context. A recent case study reported the use of Endoret^®^ PRGF in six patients with DFUs. The PRGF was injected around the wound margins, and a fibrin clot was placed over the bed of the ulcer. All six patients achieved full epithelialization of their ulcers, and mean duration of the ulcer healing was 8 weeks.^27^ While these cases may present promising results, larger‐scale studies with adequate sample size are needed to confirm the effectiveness and benefits of PRGF for DFU care.

##### Venous leg ulcer

In VLUs, a greater reduction in the area and volume was observed in sites treated with PRP compared to compression therapy (74% and 81% vs. 40% and 48%, respectively).^28^ At the 3‐, 6‐, and 12‐month follow‐ups, a significantly higher proportion of ulcers healed completely after PRP injection (85%) compared with conventional therapy (control). In a RCT including 80 patients, the healing time required for complete closure was also significantly shorter after PRP injection than treatment with nonadherent dressing and compression alone (control group).^29^ When different types of PRP delivery were compared for treatment of VLU, a superior and faster ulcer healing was observed after PRP injection followed by PRP dressing application, then compression therapy alone.^11^

In contrast, a pilot study with patients presenting with at least a six‐month history of VLU did not find difference in the mean reduction of ulcer size between PRP gel and conventional polyurethane dressing, potentially explained by the large amount of variance due to the small sample size.^30^ Despite that, additional analysis by the same author showed that VLUs treated with PRP were 4.3 times more likely to close than sites treated with the standard of care.^30^

Some studies have advocated a direct correlation between the efficacy of PRP/PRGF, the initial area of the ulcer, and its clinical course.^28^, ^31^ In VLUs with a mean area > 10 cm^2^, the treatment with autologous PRGF also resulted in greater percentage of healed areas (67.7%) compared to the application of dressing with saline (11.2%).^31^

Overall, these findings suggest that PRP/PRGF may be a more effective and a faster treatment option for chronic VLUs compared to conventional methods alone, such as dressing and compression, and should be considered during wound clinical management.

##### Pressure ulcers

The use of PRP gel has shown to accelerate wound healing,^12^, ^32^ reduce inflammation, and slow the progression of the disease in pressure ulcers by decreasing levels of inflammatory markers such as interleukin‐1β (IL‐1 β), interleukin‐8 (IL‐8), and tumor necrosis factor‐alpha (TNF‐α), as well as regulating the levels of tissue inhibitor of metalloproteinase‐1 (TIMP‐1) and matrix metallopeptidase‐9 (MMP‐9) and increasing the levels of VEGF, stromal cell‐derived factor‐1 alpha (SDF‐1α), and C‐X‐C chemokine receptor type 4 (CXCR4) in the wound region.^12^

A randomized controlled study was conducted to compare the effects of PRP gel and conventional dressing with physiological saline on the healing process of coccyx pressure ulcers in 60 patients over a period of 2 months.^33^ The sites treated with PRP gel showed a significant reduction in the area of the PU, the amount of exudate, and the necrotic/granulation tissue as assessed by the Pressure Ulcer Scale for Healing (PUSH), while no significant improvement was observed with conventional therapy.^33^ Similarly, in a study where PRP was combined with negative pressure wound therapy (NPWT), the group receiving the combination therapy showed significantly lower PUSH scores and shorter wound healing times compared to the control group receiving NPWT alone.^34^

Different types of PRP applications may have varying effects and outcomes, but research suggests that PRP, whether in gel or injected form, can effectively treat pressure ulcers, leading to better epithelization and neovascularization.^32^ However, further research is necessary to determine the optimal application method and dosage for different ulcer types and sizes.

The use of PRGFs has also been investigated for the treatment of bedsores as an adjunctive to conventional wound care or in combination with other therapeutic modalities such as hyaluronic acid. An RCT assessed the efficacy and safety of treatment of PUs ≤10 cm in 100 patients by topical application of one or two doses of PRGF alone or two doses of PRGF in combination with hyaluronic acid (HA).^35^ After 36 days of treatment, all test groups achieved a significant reduction in PU size in comparison with the standard care, which consisted of ulcer debridement and cleaning, followed by the application of liquid hydrogel and polyurethane dressing (10.3 ± 13.3%). When comparing the test groups, the greatest reduction was obtained with the application of two PRGF doses plus HA (80.4 ± 27.0%), followed by two doses of PRGF alone (54.8 ± 44.7%) and, finally, a single dose of PRGF (48.3 ± 25.8%). However, significant difference was only found between one dose of PRGF and two doses of PRGF plus HA.^35^

PRP and its derivative form, PRGF, seem to be applicable treatment options for pressure ulcers when used alone or in combination with conventional therapy, such as wound dressings and offloading techniques, and should be considered on a case‐by‐case basis considering individual patient factors, wound characteristics, and the availability of other treatment options.

#### 3.1.2 Patient‐reported outcomes (PROMs) and health‐related quality of life

Only a limited number of studies have been reported on PROMs and patient perception about therapy when using PRP/PRGF in chronic wounds.

##### Diabetic foot ulcer

One recent feasibility study by Smith et al. reported that DFU patients treated with fat grafting plus autologous PRP had higher health‐related quality of life (HRQoL) scores compared to those receiving standard podiatry wound care. However, due to the small sample size, the findings cannot be considered definitive.^36^

##### Venous leg ulcer

The use of PRP has shown favorable results in the treatment of VLUs, with both PRP gel^30^ and injection^28^, ^29^ leading to pain reduction and improvement in patients' quality of life. In combination with compression therapy, PRP has been found to further enhance clinical outcomes, resulting in significant decrease in pain visual analog scale (VAS) scores from 6.5 to 1 at 3 months and to 0.5 at 6 months post‐treatment.^28^ Moreover, a pilot study investigating the use of PRP in conjunction with light‐emitting diode therapy demonstrated significant improvements in clinical symptoms, such as pain, itching, heaviness, and leg swelling, after just 6 weeks, with 75% of patients reporting subjective improvement in their ulcers and satisfaction with the treatment.^37^

In addition, autologous PRGF was found to be more effective in reducing pain, as measured by the VAS, than saline dressings in leg venous ulcers.^31^

These findings highlight the potential of PRP/PRGF as a valuable adjunct therapy in the treatment of VLU, with the potential to improve patient outcomes and quality of life.

##### Pressure injuries

VAS score for pain severity was found to be significantly lower in patients with refractory pressure injuries, after 21 days of treatment with PRP associated with NPWT than NPWT alone.^34^ The decrease in pain may have the added benefit of reducing the need for treatments for pain control such as opioids.

#### 3.1.3 Cost implications

##### Diabetic foot ulcer

A prior computerized decision analysis using a hypothetical group of 200 000 patients has found that the average 5‐year direct wound care cost was $15,159 for PRP gel, $33,214 for saline gel, and $40,073 for standard of care. Alternative therapies, such as human fibroblast‐derived dermal substitute, allogenic bilayered culture skin substitute, bilayered cellular matrix, and negative pressure wound therapy, had varying costs from $20,964 to $47,252.^38^

Another study evaluating the cost‐effectiveness of standard care and two PRP preparation methods, commercialized (commercial kit of a gravitational platelet separation system, which contains all material necessary to extract patient's blood and prepare PRP in the same visit) and manual (PRP obtained with a “manual” laboratory procedure using a standard centrifuge technique at hospital) for DFU treatment reported that the manual method was more effective and less costly than usual care over a 5‐year period. However, PRP treatment using a commercial kit (Endoret® by BTI) was more expensive due to medical devices and nurse time.^19^ It is worth noting that this study did not differentiate between PRP and PRGF and used the same terminology for both.

The main differences in costs may be related to the number of weekly medications carried out for each of the treatment options, staff time, prices of commercial PRP/PRGF kits, time of hospitalization, recurrence, and treatment effectiveness.^19^, ^38^, ^39^ The improved healing rates expected with the PRP/PRGF could result in substantial cost savings during wound/ulcer management because of fewer complications and improved patient quality of life. Thus, in the future a potential reduction in the prices of available commercially kits could have a positive impact toward economic outcomes.

##### Venous leg ulcer

A pilot study has suggested that in the context of primary care, PRP preparation and application in VLUs may require more staff than when standard of care is provided. In addition, the time spent in applying PRP was considered longer than the required time to perform conventional treatment.^30^ This is an important point to be considered, as in hospital and clinical settings therapeutic options should also be time effective.

##### Pressure ulcer

There are still a scarce number of studies evaluating the influence of PRP/PRGF on costs of pressure ulcer treatment. A study by Uçar and Çelik (2020) found that the cost of consumables used in the PRP gel dressing group was lower than the cost of consumables in the conventional treatment (gauze dressing and saline). However, when the cost of the centrifuge device was included, the PRP group costed more than conventional treatment.^33^ It is important to note that the centrifuge device is a one‐off cost; moreover, given that the wound healing may be accelerated when PRP/PRGF is used, it can be speculated that care costs and the workload of health professionals will decrease in parallel with the shortening of hospitalization period of the patient. However, further studies are needed to confirm this hypothesis. On the other hand, it is also important to investigate how the time and staff training aiming to develop expertise on the use of PRP/PRGF could influence costs of treatment.

#### 3.1.4 Complications

##### Diabetic foot ulcer

Failure of normal wound healing in diabetic patients has been recognized to be one of the main contributors for limb amputation.^4^ Although the use of PRP accelerated DFU healing time, it did not significantly alter the need and level of limb amputation or the need for further treatments, such as graft or angioplasty, compared to the use of saline and ointment dressing.^21^ In line with this, a recent meta‐analysis has shown no differences in rates of wound complications, recurrences, or dermatitis between the PRP use and standard of care.^6^ When considering the use of PRGF on 6 patients, no adverse events or complications were observed throughout the treatment period.^27^

Additionally, it may be speculated that the repetitive collection of whole blood particularly for larger wounds/ulcers which may require multiple applications of medication/dressings could cause an additional health burden, compromising the safety and well‐being of patients. An FDA‐approved multicenter trial investigating the effects of frequent blood draws on health and safety demonstrated that PRP gel is safe for use in the treatment of nonhealing DFU and does not cause shifts in hematological and clotting factors. In addition, the study has shown that bovine thrombin usually used to activate the PRP did not cause factor V inhibition, which could lead to bleeding disorders. Similarly, the use of PRP did not affect chemistry test results for sodium, potassium, chloride, bicarbonate, creatinine, or albumin, throughout the study and into the follow‐up.^20^

##### Venous leg ulcers

In VLU cases, the overall ulcer recurrence rate after complete closure was found to be 7.8%, regardless of the treatment used, standard of care or PRP.^11^ However, neither PRP injection nor PRP dressing provided any significant benefit in terms of recurrence prevention. On the other hand, some studies have reported either no recurrence or a lower recurrence rate in VLU cases treated with PRP compared to conventional treatment. These studies suggest that PRP may have a beneficial effect on preventing VLU recurrence.^29^, ^37^ Differences in terms of adverse events between PRGF and standard care (saline plus gauze dressing) were also not found.^13^

Bacterial infection is also one of the most serious and common complications impairing wound healing and tissue repair/regeneration. In terms of microbiological changes, it was observed that hard‐to‐heal leg ulcers treated with autologous PRP had a higher bacterial contamination and percentage of bacteria compared to conventional treatment. However, this did not lead to a higher incidence of infection.^40^

Although studies suggest PRP is an effective treatment option for chronic VLUs, accelerating wound healing compared to conventional methods alone, it seems that there is conflicting evidence regarding the recurrence of VLU after treatment with this type of platelet concentrate. It is worth noting that wound healing and recurrence are complex processes influenced by numerous factors, and the optimal treatment approach may vary between individuals.

##### Pressure ulcers

When it comes to treating pressure ulcers (PUs), the addition of PRP to NPWT did not show a significant difference in postoperative complication incidence compared to NPWT alone (13.73% vs. 7.84%, respectively^34^, ^41^). However, PRP has demonstrated promising results in reducing bacterial colonization rates in PUs. For instance, the bacterial colonization rate of PUs treated with PRP decreased from 92% at the time of enrollment to 24% after 5 weeks, while sites treated with saline dressing showed a reduction from 84% to 76%.^41^ Furthermore, in a 36‐day follow‐up period, no signs of infection were observed in PUs treated with PRGF alone or in combination with hyaluronic acid, suggesting a potential antimicrobial effect of PRGF.^35^

### 3.2 Surgical wounds

Surgical wounds are among the most prevalent type of wounds, causing significant economic burden to the health system, especially when infection occurs.^5^ A surgical wound is a consequence of a specific surgical procedure performed for various reasons, including the treatment of diseases and injuries or further investigations of determined condition. Furthermore, surgical wounds may be created when part of the skin is transplanted from one area to another (skin graft).

These wounds are made in a sterile environment, where many variables can be controlled or limited such as bacteria, wound size, location, and the nature of the wound itself. They generally heal by primary closure during which the wound edges are brought together. Some types of surgical wounds, such as sternal wounds, are more difficult to heal due to their anatomical position or an increased likelihood of infection as well as those in patients presenting with underlying conditions.^42^

#### 3.2.1 Wound size and healing

##### Skin grafts (grafts and donor sites)

Several studies investigated the use of PRP in split‐thickness skin grafting (STSG). STSG is a popular technique to treat large wounds and consists of a full epidermis and a portion of the dermis harvested from a donor site, which is then left to heal on its own.^43^ The portion of the dermis that is left behind helps in the regrowth of new skin at the donor site. In this context, PRP has been tested either in the wound bed to provide immediate skin graft anchorage as well as inosculation of the STSG with a nutrient‐rich blood media or to accelerate the healing and reduce scar development in the donor site.

Overall, studies suggested that, regardless of the etiology of skin wounds, PRP can significantly shorten the healing time of sites grafted via STSG^44^, ^45^ and reduce the risk of scar hypertrophy.^46^ This is likely the result of shearing force reduction and enhancement of the wound environment with growth factors. One comparative study tested also the combination of skin flap transplantation and PRP for the treatment of open fractures and indicated a tendency for faster wound and fracture healing when the platelet concentrate was employed.^47^

When looking at the donor site, prospective and retrospective studies in patients undergoing STSG indicated that PRP gel can significantly speed up the mean wound healing time and restrict scar development, as compared to the use of a petrolatum gauze or paraffin gauze dressing.^48^, ^49^, ^50^ Remarkably, a study comparing the use of PRP (proximal half) or paraffin gauze (distal half) within the same donor site indicated a faster healing induced by PRP up to 14 days, while on day 21 similar outcomes were observed in the two groups.^51^ Furthermore, Guerid et al.^52^ have shown that adding an autologous keratinocyte concentrate to the platelet concentrate can further accelerate the healing time at the donor site. Another recent RCT on dermo‐epidermal grafts reported that PRP at the donor site reduced the wound healing time by a mean of 17.8% (14.9 days vs. 18.4 days).^53^

To the best of our knowledge, no studies have employed PRGF in association with skin grafts. Thus, further studies are required to investigate whether this subtype of PRP with a more sustained release of growth factors could offer superior results over conventional PRP in terms of healing time/rate and scar reduction.

##### Surgical incisions

Two RCTs evaluated the efficacy of PRP on cesarean sections' wounds. In particular, after closure of the fascia and prior to skin closure, PRP was directly applied to the subcutaneous tissue of the wound site by using a sterile syringe.^54^, ^55^ According to the Vancouver Scar Scale, PRP application had a significant benefit beginning on the 5th^54^ day or 7th^55^ day after surgery, and the trend was still visible at 6 months post‐surgery.^55^ Moreover, the PRP group showed a greater reduction in the Redness, Edema, Ecchymosis, Discharge, Approximation (REEDA) score compared to the control group,^54^, ^55^ with better cosmetic appearance and proper wound coaptation compared to the control group.

A recent study evaluated the esthetic outcomes of using PRP after plastic and reconstructive maxillofacial surgeries.^56^ Fifty patients were injected PRP intradermally after suturing the wound, and the platelet concentrate was also applied over the wound, while fifty patients did not receive PRP. The most significant differences were noticed at the 10th and 30th days, where scar width was significantly smaller in the PRP‐treated subjects.

**FIGURE 2:**
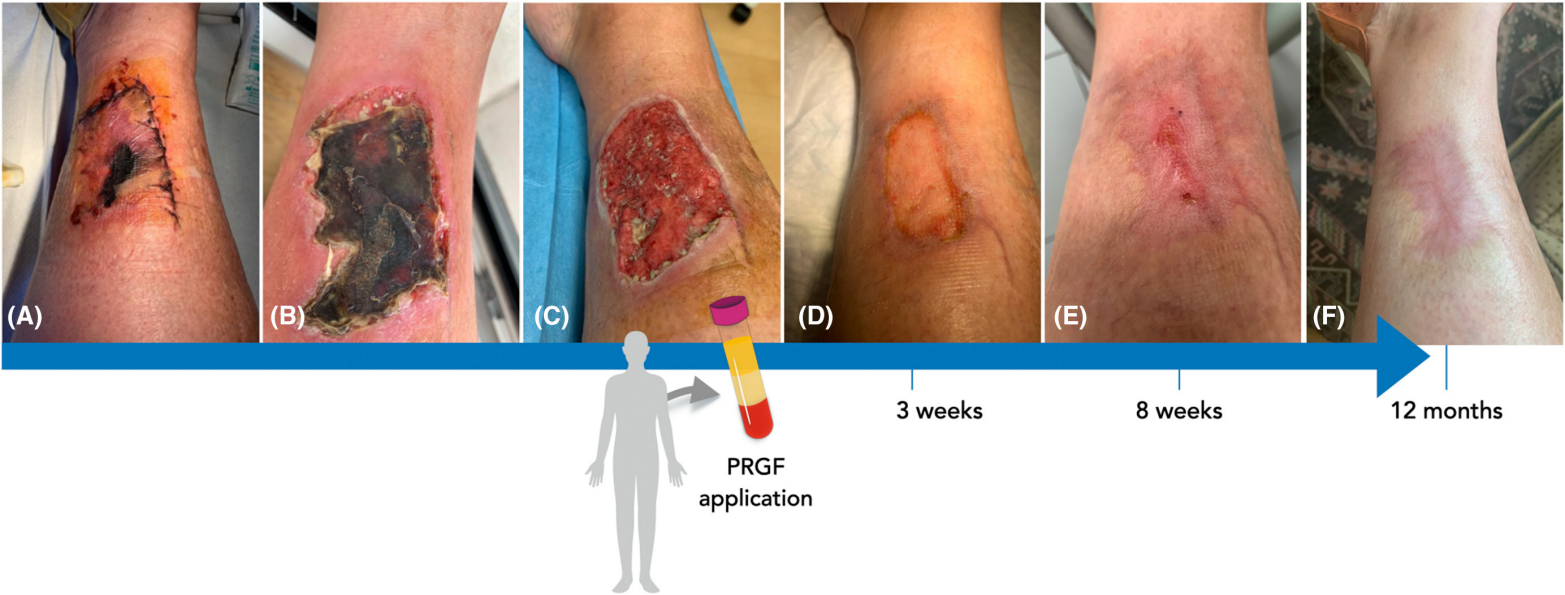
Clinical case on the application of plasma rich in growth factors (PRGF). (A) A healthy 55‐year‐old woman presenting with a leg wound due to an accidental fall while cycling underwent an early flap surgery. (B) Two to five days after the surgical procedure, the flap showed signs of necrosis, (C) resulting in an open wound (10 x 6 cm). Topical treatment with PRGF Endoret‐Serum applied every 2 days at a homecare basis was advised as minimally invasive therapeutic option to promote healing. (D) After 3 weeks of treatment, healthy granulation tissue and rapid epithelization were observed. (E) The treatment with Endoret‐Serum continued for 8 weeks as the wound showed signs of full epithelization with low edge inflammation and minimal scar development. No wound infection was observed, and the treatment with Endoret‐Serum was concluded. (F) At 12‐month follow‐up, the treated area presented healthy cutaneous tissue appearance. Clinical case courtesy of Dr. Eduardo Anitua.

Despite adequate care, some surgical procedures such as graft and surgical flaps may present impaired healing and vascularization leading to tissue necrosis and open wound, requiring further treatment. Figure 2 exemplifies a case of a healthy 55‐year‐old woman who presented with a leg wound due to an accidental fall while cycling. The patient underwent an early flap surgery; however, 2–5 days after the surgical procedure, flap showed signs of necrosis resulting in an open wound (10 cm x 6 cm). Although standard wound care, including debridement and cleaning with saline was provided, healing was not achieved within the expected time. Therefore, the topical treatment with PRGF Endoret^®^‐Serum was advised aiming to promote tissue healing through a minimally invasive therapeutic option. Six weeks prior to the beginning of treatment with PRGF, patient underwent topical and systemic antibiotic therapy to eliminate any potential infection. PRGF Endoret^®^‐Serum was applied every 2 days at a homecare basis. After 3 weeks of treatment, healthy granulation tissue and rapid epithelization were observed. The treatment with Endoret^®^‐Serum continued for 8 weeks as the wound showed signs of full epithelization with low edge inflammation and minimal scar development. No wound infection was observed, and the treatment with Endoret^®^‐Serum was concluded. At 12‐month follow‐up, the treated area presented healthy cutaneous tissue appearance.

#### 3.2.2 PROMs and health‐related quality of life

##### Skin grafts (grafts and donor sites)

By shortening the healing time of STSG, PRP allows to reduce the number of dressing changes at the recipient site and the need for drains and pressure dressing, which are the main causes of discomfort for the patient, thus also reducing the overall operative time.^44^, ^45^ An RCT in 200 patients receiving STSG confirmed that the use of PRP in the recipient site reduced the frequency of dressing and the mean hospital stay.^46^

In a study on exposed fractures, Wang et al.^47^ reported that the 36‐Item Short Form Survey ([SF‐36] physiological function, role limitations due to physical health problems, somatic pain, and overall health) of patients that received PRP together with a skin transplant were significantly higher than the patients that did not receive PRP. According to the patient and observer scar assessment scale (POSAS), healing was significantly better in the PRP group at 30 days, with a mean POSAS of 2.5 ± 0.1, as compared to a mean POSAS of 5.8 ± 0.1 in the control group. Similar outcomes were reported at 90 days.

A retrospective study comparing PRP with a petrolatum gauze dressing at the donor sites of STSGs failed to report differences in pain intensity at 3 and 21 days.^48^ However, at 7, 10, and 14 days postoperatively, the pain intensity during the dressing process was significantly lower in the PRP group. A similar result at 7 days was also reported by Miller et al.^57^

The beneficial effect of PRP on patient's pain experience was recently confirmed in two prospective controlled studies that indicated significantly less pain in the group that received PRP instead of a paraffin gauze or petroleum gauze dressing at the donor site.^49^, ^50^ Likewise, another study comparing the use of PRP (proximal half) or paraffin gauze (distal half) within the same donor site indicated a significant reduction in the severity of pain and pruritis where PRP was applied.^51^ Remarkably, one study suggested that adding an autologous keratinocyte suspension to the platelet concentrate further reduced pain experience at the donor site.^52^

##### Surgical incisions

PRP added intradermally and over surgical wounds following plastic and reconstructive maxillofacial surgeries was associated with improved dermatological quality of life index (DQLI).^56^ In particular, a 4 times improved score was recorded at 30 days post‐surgery and a similar outcome was also reported at 90 days.

In an RCT examining the effect of PRP on postoperative sternal wound healing in patients receiving cardiovascular surgery, Englert et al.^58^ reported reduced chest and leg pain when PRP was employed.

Likewise, an RCT showed that applying PRP to cesarean sections' wounds led to reduced pain, based on VAS scores (42% reduction vs. 31% reduction at 5 days and 51% reduction vs. 48% reduction after 8 weeks).^54^ This outcome was confirmed by another RCT, which showed improved VAS up to 6 months post‐surgery.^55^

#### 3.2.3 Cost implications

##### Skin grafts (grafts and donor sites)

No structured economic assessments were reported in the identified studies. However, Gupta et al.^59^ reported that the overall cost of PRP as a preparative for resurfacing burn wounds STSG was significantly lower than staples and sutures' costs (200–300 rupees vs. 2000–3000 rupees). Likewise, the same group indicated PRP as a cheaper option as compared to other dressings for the donor site such as negative pressure wound therapy, collagen, or hydrofiber.^49^

##### Surgical incisions

In a meta‐analysis assessing the use of PRP to prevent sternal wound infections following cardiac surgery, none of the included studies provided robust cost analyses.^60^ However, the authors calculated, from the odds of developing sternal wound infection, that the number of patients who would need to be treated with PRP to prevent one case of mediastinitis was 140 (95% confidence interval: 73.1–1506.2). As such, considering the overall cost of deep sternal wound infections (estimated to be $300,000), the cost of platelet gel for 140 patients ($84,000) would still favor the prophylactic use of PRP.

A large study on 2000 patients reported a significant reduction in the overall actual cost in the total management of deep and superficial sternal wounds when PRP was employed ($1,256,960 to $593,791 respectively).^61^ Despite the significant overall reduction in cost in the management of sternal wounds, the number of patients needed to be treated to see a benefit was 71 (95% confidence interval [CI]: 41.8 to 244.5), with a cost of $27 000 to prevent one deep sternal wound infection and cost break‐even point. However, with superficial sternal wounds the number of patients needed to be treated to see a benefit was 17 (95% CI: 12.7 to 24.3), with a cost of $6,417 to prevent one infection, but a cost break‐even point did not exist. The overall number of patients needed to be treated to see a benefit was 14 (95% CI: 10.5 to 18.9), with a cost of $5,203 to prevent one overall wound infection and cost break‐even point. More recently, in a study in obese diabetic patients it was estimated that the PRP‐associated cost to reduce the risk of sternal wound complications was €3,875−€3,630 for each avoided complication.^62^

#### 3.2.4 Complications and recurrence

##### Skin grafts (grafts and donor sites)

Based on 4 RCTs, a systematic review indicated that PRP decreased the odds of graft loss in STSG procedures by 85% and the odds of hematoma formation by 79%.^63^ A comparative study on open fractures also indicated that adding PRP to a skin transplant led to a reduced rate of infections (2.7% vs. 11.4%).^47^ While PRP is routinely produced from the patient's own blood, one study reported favorable outcomes when using an allogenic PRP in association with STSG. When the platelet concentrate was applied, a 100% graft uptake was reported, while 4 out of 20 cases where it was not applied had complete graft loss.^64^

Regarding the recipient bed, a RCT concluded that PRP helped fixating the split skin graft and helped preventing seroma formation and other complications.^65^

##### Surgical incisions

Several studies investigated the use of PRP to prevent sternal wound infections following cardiac surgery. A meta‐analysis considering both prospective and observational studies concluded that PRP may significantly reduce the odds of developing sternal wound infections, including mediastinitis, while there is no evidence of reducing bleeding complications.^60^ However, it should be noted that quality of the evidence was poor and that observational studies rather than RCTs were more likely to report on a significant benefit.

A large study on 2000 patients over a 7‐year period not included in the aforementioned meta‐analysis indicated that PRP reduced the incidence of deep sternal wound infections from 2.0% to 0.6%, superficial wound drainage from 8.0% to 2.0%, and hospital readmission rate within 30 days of operation from 4.0 to 0.8.^61^ Interestingly, the time to the infection post‐surgery demonstrated that all infections in the PRP group occurred within the first 2 months post‐surgery, whereas in the control group they occurred up to 4 months post‐surgery.

More recently, another systematic review and meta‐analysis confirmed that retrospective cohort studies clearly indicate a significantly reduced incidence of deep sternal wound infection when using PRP, while RCTs overall fail to confirm this outcome.^66^

Remarkably, a study using a historical control cohort suggested a beneficial effect of PRP in reducing the risk of deep sternal wound problems in high‐risk obese diabetic patients (from 11% to 4.2%).^62^

In conclusion, the available studies suggest that PRP may accelerate the mean wound healing time and limit scar development in surgical wounds. As such, PRP appears to positively influence PROMs, mainly by reducing the pain experienced, and is reported to be a cost‐effective treatment by the few studies that indicated a reduced incidence of complications. However, future studies accounting for confounding variables (including use of medication for pain control, associated therapies, and comorbidities) are warranted.

Only limited evidence is available for PRGF; therefore, no recommendations can be made on its use in surgical wounds.

### 3.3 Burn

Burn injury is a trauma to the skin or underlying tissue mainly caused by thermal, chemical, radiation, and electrical energy exposures.^67^, ^68^, ^69^ It is an acute wound that in several cases becomes chronic and^70^ whose disturbance of epidermal–mesenchymal interactions due to delayed epithelialization leads to the development of fibrotic conditions.^71^, ^72^

Burns can result in either shallow wounds or deep wounds, which may be an important factor for the prognosis and quality of repair.^73^ Shallow wounds heal in a shorter period if the blister wall is maintained intact and re‐epithelize within 2 to 3 weeks.^70^, ^74^, ^75^ On the other hand, deep wounds often require operative management. Furthermore, the severity of the burn depends on the percentage of the total body surface area (%TBSA) involved. Johnson et al.,^68^ in their review, defined severe wounds as those involving >15% of the TBSA.^68^

The choice of an appropriate therapeutic approach for burn wounds requires evaluation of the percentage of the TBSA, the depth of the defect, and the patient's systemic condition.^68^ Treatment of burn wound imposes early excision of the damaged tissue and immediate wound closure. Various studies evaluated the use of PRP on burn wounds; however, its clinical potential is not completely clarified, mainly due to the different pathophysiological characteristics of burn wounds, including greater edema, decreased perfusion, and microthrombus formation.^67^ Despite a recent study that investigated the use of a storable topical serum based on PRGF in promoting burn wound healing in 3D skin models, there is still a lack of clinical studies evaluating the use of this specific derivative of PRP.

Autologous STSG is the primary mode of wound closure for major burns, although scarring and wound contraction are among the main disadvantages (see section 3.2.1.1: Skin grafts (grafts and donor sites)).^76^ In addition, donor site management is critically important particularly in the severe cases.^77^

#### 3.3.1 Wound size and healing

##### PRP alone

In the early 2000s, patients with wide friction burns in the femur, among others, were included in a prospective RCT aiming to evaluate the effect of PRP gel application. The results showed that the use of PRP gel led to faster healing rates and adequate tissue regeneration, while the area to be covered with skin graft was reduced to 35.6% (range: 23%–49%) of the initial wound measurement.^78^ Furthermore, a randomized double‐blind controlled trial found that the platelet dressing repeatedly applied on the burn wound reduced healing time.^79^ On the contrary, a prospective study found PRP use did not significantly improve wound healing or scar formation in acute burns.^80^

Two recent systematic reviews^67^, ^73^ revealed significant differences in the healing rate, healing time, and scar assessment score between PRP‐treated wounds and those in which other treatments were applied, while no significant difference in the degree of epithelialization was observed. Despite the results, the authors characterized the level of evidence as low due to the small number of studies and the significant variations in the study design, methodology, follow‐up periods, PRP preparation, and wound type. Furthermore, another systematic review aiming to assess the efficacy of PRP on burn wound healing, measured time to complete epithelialization and rate of wound closure by the end of weeks 2 and 3.^81^ The authors concluded that PRP accelerated burn wound closure compared to conventional dressings and placebo.

The application of a lyophilized PRP (LPRP) powder on deep second‐degree burn wounds has also been investigated, showing better wound closure at 3 weeks in the LPRP group compared to the control group without the PRP supplement.^69^ The LPRP powder, manufactured by vacuum freeze‐drying and gamma‐ray sterilization, presents good thermostability, facilitates platelet storage, exhibits continuous release of growth factors, reduces contamination rate, and increases shelf life.

The application of autologous platelet‐rich gel (APG), prepared by mixing up PRP, thrombin, and calcium chloride, was evaluated in patients with deep grade II burn wounds.^82^ The results showed that the wound healing time, the ratio of healed area, and the frequency of dressing changes in the treatment group were significantly lower than those in the control group, indicating the clinical potential of APG on wound healing, which might be due to the increased concentration of local growth factors.

##### PRP plus skin graft

The combination of PRP and skin graft for the reconstruction of burn wounds showed enhancement of viscoelastic properties of skin areas and a modest amelioration of the healing time.^83^ In a clinical study, five out of 20 cases with 1–4‐year‐old postburn contracture were treated with PRP. PRP was applied on half of the wound before the placement of the skin graft, while the other half was served as control with no PRP application. The results were contradictory showing either poor healing or better healing compared to the control areas.^64^

Additionally, it has been shown that the use of PRP associated with skin graft, contributed to secure the graft to the wound bed and enhanced healing in patients with well‐controlled systemic diseases.^84^ The findings from another study also demonstrated that wound areas treated with STSG plus autologous platelet concentrate (Harvest® SmartPrep® Platelet Concentrate System, Harvest Technologies Corporation, Plymouth, MA, USA) retrieved quicker the viscoelastic properties of normal areas than those areas treated with STSG alone.^85^

In the study of Prochazka et al., 18 patients with second‐ or third‐degree burns were included and were treated with autologous dermo‐epidermal skin grafts (DESGs) and APC (Harvest® SmartPrep® Platelet Concentrate System, Harvest Technologies Corporation, Plymouth, MA, USA). An increase in the percentage of healed area was observed, with >94% of patients having over 99% of the burned area healed 18 days after the procedure. The results of this study showed that the patients had high quality of healing without evidence of scar hypertrophy, while 72% of the grafts were re‐epithelialized by day 10, thus generating a barrier to infection and other complications.^86^

In contrast, a randomized controlled clinical study found that the addition of autologous leukocyte containing PRP to a STSG in the treatment of deep dermal to full‐thickness burn wounds did not result in improved graft take (percentage of the graft that was vital and showed good adherence to the wound bed) and epithelialization rate when compared to control wounds treated with STSG alone. The authors underlined the conflicting evidence of PRP effect among the studies, the diversity of PRP products, and their preparation.^80^

#### 3.3.2 PROMs and health‐related quality of life

##### PRP alone

The application of autologous platelet‐rich gel (APG), prepared by mixing up PRP, thrombin, and calcium chloride versus Silvadene cream by external application (control), was evaluated in patients with deep grade II burn wounds.^82^ On the 7th day, the VAS and the Vancouver Scar Scale (VSS) scores in the treatment group were significantly lower than those in the control group, indicating that APG had obvious better effect on alleviating the pain, wound healing, and unapparent scar hyperplasia.^82^

##### PRP plus skin graft

Out of 18 patients with second‐ or third‐degree burns treated with autologous dermo‐epidermal skin grafts (DESGs) and spray‐coated with APC (Harvest® SmartPrep® Platelet Concentrate System, Harvest Technologies Corporation, Plymouth, MA, USA), 78% required analgesics prior to surgery, and this percentage decreased to 6% of patients by 14th postoperative day. The monitoring of the pruritus by the visual analog pruritus scale (VAPrS) showed that 94% of the patients at postoperative day 4 did not require antihistamines and the percentage remained at low levels (78%) throughout the observation period (up to 12 months).^86^

#### 3.3.3 Cost implications

##### PRP alone

As previously mentioned, when discussing skin grafts in a clinical study aiming to evaluate the use of PRP over difficult burn wound beds to augment graft uptake and attenuate complications, 200 patients with burns and healing ulcers were included.^59^ It is worth noting that the exact number of patients with burn wounds is not referred. PRP was used just before the application of skin grafts, while in the control group, the patients underwent grafting by the standard method. In the PRP group, there was a benefit in terms of the cost.

##### PRP plus skin graft

Despite the lack of studies accessing the cost implications of PRP use for treatment of burn wounds, a study by Prochazka et al. (2014)^86^ revealed that although DESG combined with APC (Harvest® SmartPrep® Platelet Concentrate System, Harvest Technologies Corporation, Plymouth, MA, USA) required longer operating times, the cost of hospital stay was lower (approximately 25% less) than that of institutional controls.^86^

#### 3.3.4 Complications and recurrence

##### PRP alone

The most important complications associated with burns are infection (burn wound sepsis) and proliferative scarring.^70^ Two recent systematic reviews revealed that although PRP use had a positive impact on the healing rate, healing time, and scar assessment score, no significant difference between PRP‐treated and no PRP‐treated wound areas was observed for graft take, infection, and incidence of adverse effects.^67^, ^73^

##### PRP plus skin graft

In a prospective randomized controlled study involving systemically compromised patients, topical application of PRP on wound beds before graft placement and anchorage was compared to the use of conventional techniques for graft fixation in the control group.^46^ The study found that the graft in the PRP group exhibited immediate adherence, while this was not observed in the control group. Additionally, only 10% of patients in the PRP group experienced graft edema and 4% developed hematoma. In contrast, the control group had a higher incidence of graft edema lasting over a week (68%) and hematoma (15%). Furthermore, all objective parameters, including hematoma, discharge from the graft site resulting in significant graft loss, graft edema, frequency of dressings, and duration of stay in the plastic surgery unit, showed statistically significant differences between the control and PRP groups. As previously mentioned in the section PRP plus skin graft, in a more recent study involving 200 patients with burns and healing ulcers, the combination of skin graft and PRP demonstrated significantly higher graft uptake rates on day 2 (88.9 ± 34.5) compared to the control group (42.5 ± 31), where patients underwent grafting using the standard method.^59^ The study also highlighted the benefits of PRP in terms of reducing hematoma formation and infection. Overall, the application of APG, prepared by mixing up PRP, thrombin, and calcium chloride, did not increase the occurrence of various adverse reactions, suggesting that it is safe in clinical practice.

In patients with burns, disorder of the hemostatic and hematological parameters may be observed, requiring an adequate assessment before PRP therapy is applied. A retrospective observational study in a large population showed that the platelets depict the lowest counts 3 days after burn and the highest counts 15 days after burn, followed by a temporary thrombocytosis, which gradually returns to normal values on day 24.^87^ Concerning the quality of PRP, it was found that, despite the effect of the burn on the hematological status, platelets are functional and not excessively activated and PRP has comparable levels of growth factors to that of matched healthy volunteers.^88^

### 3.4 Other complex extra‐oral wounds

#### 3.4.1 Ocular surface and corneal disease

Neurotrophic keratitis (NK) also known as neurotrophic keratopathy is a rare degenerative corneal disease characterized by reduction or absence of corneal sensitivity and consequent dysfunction of corneal healing (i.e., stromal ulcers) process followed by irreversible visual deficit.^89^, ^90^ NK can be caused by different conditions including infectious (herpes simplex, herpes zoster, and leprosy) and congenital (i.e., ectodermal dysplasia) factors, physical injuries, and systemic diseases (i.e., diabetes mellitus and autoimmune disease).^89^ The treatment of NK is extremely difficult and aims to improve the condition of corneal epithelium while also preventing the development of corneal ulcers and their subsequent perforation.

A pilot study investigated the use of PRP daily eye drops along with preservative‐free artificial tears and vitamin A ointment for 3 months in patients with neurotrophic corneal ulcer due to herpes zoster/simplex infection or injury to the trigeminal and/or facial nerve. The results showed improved visual acuity and less subjective symptoms in all patients. Complete healing of the ulceration was observed in 80% of patients, and no side effects were reported.^89^, ^91^ Similar results were obtained when patients diagnosed with NK stages 2 (persistent epithelial defect) and 3 (corneal ulceration) were treated with PRGF (Endoret®) eye drops. The complete resolution of the defect/corneal ulcer took an average of 11.4 weeks (SD = 13.7), and it was achieved in 97.4% of eyes assessed. After treatment, there was a significant reduction in the ocular surface disease index (OSDI; 61%), VAS for frequency and severity (60%) of ocular symptoms (i.e., discomfort/pain, dryness, burning, photophobia, foreign body sensation, blurred vision, and itching), and improvement of best‐corrected visual acuity (BCVA; 53%).^90^

A retrospective study on 74 patients, affected by different ocular surface and corneal diseases with associated ulcers, investigated the use of PRGF along with a regenerating agent matrix (RGTA) eye drop therapy (Cacicol20, OTR3, Paris, France) in cases where ulcer closure was not initially achieved by RDTA application alone. This study found that PRGF was used for ulcer closure in 96.2% of eyes with a mean treatment time of 4.2 ± 2.2 (1.5–9.0) months. PRGF eye drops significantly reduced the percentage area of the corneal defect as well as corneal staining. BCVA, VAS (frequency and severity of ocular symptoms), and OSDI improved from the baseline, and intraocular pressure (IOP) remained unaffected.^92^ In summary, these studies suggest that the use of PRP or PRGF eye drops, along with other supportive measures, has potential in promoting corneal healing, reducing symptoms, and improving visual outcomes in patients with these specific conditions. However, further research and larger clinical trials are required.

#### 3.4.2 Leprosy ulcer

Treatment of leprosy comprises the treatment of the disease per se and the alleviation of the complications to successfully ameliorate patient's quality of life.^89^ A variety of therapeutic nonsurgical (such as saline or collagen dressings and topical application of metronidazole, growth factors, and PRP) and surgical approaches have been performed to improve the healing of ulcers due to leprosy.^89^ In a prospective interventional study, an attempt was made to determine the effectiveness of PRP in the healing of trophic ulcers. The findings proved that the application of PRP greatly reduced the duration of treatment and shortened the hospital stay improving the patient's quality of life.^93^ Furthermore, in a randomized controlled trial, PRP combined with total contact casting was compared with total contact casting alone for treatment of trophic ulcers in leprosy.^89^ Τhe size and surface area of the ulcer were significantly improved in the combined PRP therapy group. The active treatment with PRP lasted only for 8 weeks, while the improvement continued for another 4 weeks without treatment.

## 4 CONCLUSIONS AND FUTURE PERSPECTIVES

The literature suggests that PRP may be an attractive strategy in different clinical scenarios for the treatment of different extra‐oral wounds, given the capability to accelerate healing, overall cost‐effectiveness, safe nature of therapy, and potential benefits to patient's quality of life. However, the findings regarding remission and complications are still heterogenous. The difference in the results could be explained by the large heterogeneity of protocols described for PRP preparation and various time points considered for assessment, as well as modes of application, with some studies using gels, injections,^11^, ^24^, ^28^ or sprays.^12^ In recent years, PRGF was introduced with a very structured and well‐defined protocol compared to PRP. Although PRGF has been used in different medical fields with promising results, literature is still limited, and larger studies are required.

To assess the clinical efficacy of PRP/PRGF on extra‐oral wound care, different predictors of healing have been used, including the number of wounds achieving complete healing, that is, 100% re‐epithelialized, change in wound size (area and/or volume), time in days to complete healing, and healing rate over the study period.^21^, ^36^, ^94^ While the impact of wounds on patient's quality of life can be significant, by affecting their ability to engage in daily activities, limiting their mobility and independence, and even leading to social isolation, currently there are fewer studies examining the impact of PRP/PRGF use on patient‐reported outcomes to draw significant conclusions. Nevertheless, some studies suggest that PRP/PRGF treatment can have a positive effect on patients' overall well‐being and daily lives, emphasizing the importance of considering patient perspectives when evaluating wound healing treatments.

Besides its clinical effects and potential influences on patients' quality of life, the wound treatment, particularly the “hard‐to‐heal” wounds, is time‐consuming and also represents a substantial economic burden for the patient and the healthcare system.^39^ Although the number of studies investigating the clinical implications of PRP and PRGF use has increased over the years, only a few of them have evaluated the relationship between the costs and clinical outcomes of treatment.^19^, ^33^, ^38^, ^39^ The costs for wound treatment usually include cost per staff (i.e., nurse and auxiliary nurse), the number of treatments or PRP/PRGF application, dressings, and the costs of the materials necessary to prepare PRP/PRGF.^30^, ^33^, ^36^ For PRP/PRGF, there is an initial investment in terms of training and purchase of centrifuges and kits, but in the long term it may be possible to recover the money and reduce the overall cost of the procedures. However, it is still not clear how many patients need to be treated before the PRP/PRGF treatment becomes cost‐effective.

While some wounds will heal within a reasonable period of time with optimal care, others will not, increasing the risk of complications^20^ and infection. Failure of normal wound healing in patients with underlying conditions such as diabetes has been recognized to be one of the main contributors to limb amputation.^4^ Bacterial infection is also one of the most serious and common complications impairing wound healing and tissue repair/regeneration. The combination of proteolytic enzymes, bacterial exudates, and chronic inflammation can alter the expression of growth factors, thus affecting the cellular apparatus needed for cell proliferation and wound healing. Overall, studies report a low risk of adverse events (i.e., fever, edema, pain, skin itching, rash, or other sensory abnormalities) related to the PRP use.^26^, ^27^, ^40^ However, cephalic vein phlebitis has been reported as one of the potential events directly related to venipuncture from taking patient blood for PRP preparation.^40^

Despite their benefits, it is essential to keep in mind that any treatment, including the use of autologous platelet concentrates (APCs), should be applied alongside conventional wound care and disease management to ensure a positive clinical outcome and enhance the healing process. Most available studies on extra‐oral wound care compare the use of PRP/PRGF to conventional treatment, also called standard of care, which ranges from wound debridement, cleaning with saline, and use of different dressings (i.e., cloth/gauze and hydrogel) to treatments such as NPWT, skin grafts, and compression therapy, depending on the type and etiology of the wound. Fewer studies compared the use of PRP with other alternative strategies to enhance wound healing or the potential benefit of different APC generations. In addition, there is still scarce evidence if PRP/PRGF used in challenging presentations (i.e., severe ischemia, exposed tendon or bone, and advanced ulcer stage) and in high‐risk population with multiple comorbidities could still assist healing and present the same results. Future studies should consider stratification of the randomization by ulcer/wound size and treatment of groups with challenging manifestations.

Some of the current PRP/PRGF systems used may require a specialized team to perform the necessary procedures, which may present higher costs for equipment. Thus, further development of smaller point‐of‐care system technologies which are less expensive, more user‐friendly, and reliable to be used in different clinical settings is warranted. Likewise, the use of novel technologies to estimate wound area and to assess the progress of healing should be considered to identify early signs of complications and standardize scientific reporting. A recent consensus has suggested that efforts should also be made to simplify the treatment of chronic wounds driving care out of the hospital and toward outpatient/home settings, where it can be delivered mainly by the patient and their family.^2^

In summary, the use of PRP/PRGF in wound care should be considered on a case‐by‐case basis, considering the type and severity of the wound, as well as individual patient factors and experience of the clinical team delivering the treatment. It is also important to highlight that standardization in reporting the use of PRP and PRGF (i.e., centrifugation protocol and type of device used) is crucial to make studies comparable and to be able to draw more meaningful conclusions on the efficacy of these products. Thus, further research is needed to determine the optimal protocols and modes of application of PRP/PRGF, which may help to elucidate the best treatment options to enhance healing, while also improving patients' quality of life in a cost‐efficient manner.

## FUNDING INFORMATION

This study received no specific grant from any funding agency in the public, commercial, or not‐for‐profit sectors.

## CONFLICT OF INTEREST STATEMENT

All authors declare no conflict of interest.

## Supporting information


Appendix S1.

## Data Availability

The data that support the findings of this study are available in the Supplementary Material of this article.
