# Supplementary material for: Platelet‐rich plasma and plasma rich in growth factors in extra‐oral wound care
Source: Periodontol 2000. 2024 Jul 26;97(1):320–41. doi: 10.1111/prd.12572 (PMC11808476; doi:10.1111/prd.12572)
Supplement: Supplementary file 1 — Appendix S1. [file PRD-97-320-s001.docx]

**SUPPLEMENTARY INFORMATION**

**Platelet-Rich Plasma and Plasma Rich in Growth Factors in**

**Extra-oral Wound Care**

Jeniffer Perussolo^1^, Elena Calciolari^1,2^, Xanthippi Dereka^3^, Nikolaos Donos^1^

**Running title:** PRP and PRGF in extra-oral wound care

**Author’s affiliation:**

^1^Centre for Oral Clinical Research, Institute of Dentistry, Faculty of Medicine and Dentistry, Queen Mary University of London, London, UK, Turner Street, E1 2AD.

^2^Dental School, Department of Medicine and Surgery, University of Parma, Parma, Italy

^3^Dental School, Department of Periodontology, School of Dentistry, National and Kapodistrian University of Athens, Athens, Greece.

**Keywords**: wound, healing, extra-oral, platelet-rich plasma, plasma-rich in growth factors

**Correspondence**

Professor Nikolaos Donos

Centre for Oral Clinical Research

Institute of Dentistry

Faculty of Medicine and Dentistry

Queen Mary University of London (QMUL)

Turner Street, London E1 2AD, UK

e-mail: n.donos@qmul.ac.uk

ORCID 0000-0002-4117-9073

**Supplementary information 1**  - Search strategy

1. Search strategy - Medline via OVID

Ovid MEDLINE(R) and Epub Ahead of Print, In-Process, In-Data-Review & Other Non-Indexed Citations, Daily and Versions <1946 to January 13, 2023>

|  |  | **MeSH terms** | **Free text** |
| --- | --- | --- | --- |
| **P** | Patients presenting extra-oral wound such as diabetic ulcers, pressure ulcers, other ulcers, burn wound or skin wounds. | diabetic Foot  foot ulcer  exp skin ulcer  exp burns  wounds and injuries  exp wound healing  skin transplantation  graft rejection | diabetic feet OR diabetic foot OR  diabetic ulcer* OR foot ADJ3 ulcer*  OR plantar ulcer*  (skin OR leg) ADJ3 ulcer* OR pressure ulcer* OR bedsore*  ulcer* OR venous ADJ2 ulcer* OR varicose ulcer*  burn*  wound*  skin graft* OR graft rejection |
| **I** | Treatment with Platelet-Rich Plasma (PRP) or Plasma Rich in Growth Factors (PRGF) | Platelet-Rich Plasma | Platelet-Rich Plasma OR PRPPlasma Rich in Growth Factors OR PRGF |
| **C** | All possible comparisons among interventions included, including non-intervention or treatment | n/a | n/a |
| **O** | Wound size, healing rate, patient-reported outcome measures (PROMS), health-related quality of life, pain management (including discomfort, painkillers intake), complication/failure of procedure (i.e., presence of infection; limb amputation) | n/a | n/a |
| **S** | Human studies | Exp animals/not humans.sh | n/a |

1. Search strategy - Embase

|  |  | **MeSH Terms** | **Free text** |
| --- | --- | --- | --- |
| **P** | Patients presenting extra-oral wound such as diabetic ulcers, pressure ulcers, other ulcers, burn wound or skin wounds. | “diabetic Foot”/de  “foot ulcer”/de  “skin ulcer”/ exp  Burn/exp  Wound/exp  “skin transplantation”/exp  “skin graft rejection”/exp | “diabetic feet” OR “diabetic foot” OR “diabetic ulcer*” OR foot NEAR/3 ulcer* OR “plantar ulcer*”  (skin OR leg) NEAR/3 ulcer* OR “pressure ulcer*” OR bedsore* OR ulcer* OR venous NEAR/2 ulcer* OR “varicose ulcer*”  burn*  wound*  “skin graft*”OR  \|”graft rejection” |
| **I** | Treatment with Platelet-Rich Plasma (PRP) or Plasma Rich in Growth Factors (PRGF) | Platelet-Rich Plasma | “Platelet-Rich Plasma” “Platelet Rich Plasma” OR PRP“Plasma Rich in Growth Factors” OR PRGF |
| **C** | All possible comparisons among interventions included, including non-intervention or treatment | n/a | n/a |
| **O** | Wound size, healing rate, patient-reported outcome measures (PROMS), health-related quality of life, pain management (including discomfort, painkillers intake), complication/failure of procedure (i.e., presence of infection; limb amputation) | n/a | n/a |
| **S** | Human studies | NOT ([animals]/lim NOT [humans]/lim) | n/a |
